# Perisomatic ultrastructure efficiently classifies cells in mouse cortex

**DOI:** 10.1038/s41586-024-07765-7

**Published:** 2025-04-09

**Authors:** Leila Elabbady, Sharmishtaa Seshamani, Shang Mu, Gayathri Mahalingam, Casey M. Schneider-Mizell, Agnes L. Bodor, J. Alexander Bae, Derrick Brittain, JoAnn Buchanan, Daniel J. Bumbarger, Manuel A. Castro, Sven Dorkenwald, Akhilesh Halageri, Zhen Jia, Chris Jordan, Dan Kapner, Nico Kemnitz, Sam Kinn, Kisuk Lee, Kai Li, Ran Lu, Thomas Macrina, Eric Mitchell, Shanka Subhra Mondal, Barak Nehoran, Sergiy Popovych, William Silversmith, Marc Takeno, Russel Torres, Nicholas L. Turner, William Wong, Jingpeng Wu, Wenjing Yin, Szi-chieh Yu, H. Sebastian Seung, R. Clay Reid, Nuno Maçarico da Costa, Forrest Collman

**Affiliations:** 1 Allen Institute for Brain Science, Seattle, WA USA; 2 University of Washington, Seattle, WA USA; 3 Princeton Neuroscience Institute, Princeton University, Princeton, NJ USA

**Keywords:** Cellular neuroscience, Computational neuroscience

## Abstract

Mammalian neocortex contains a highly diverse set of cell types. These cell types have been mapped systematically using a variety of molecular, electrophysiological and morphological approaches^1–4^. Each modality offers new perspectives on the variation of biological processes underlying cell-type specialization. Cellular-scale electron microscopy provides dense ultrastructural examination and an unbiased perspective on the subcellular organization of brain cells, including their synaptic connectivity and nanometre-scale morphology. In data that contain tens of thousands of neurons, most of which have incomplete reconstructions, identifying cell types becomes a clear challenge for analysis^5^. Here, to address this challenge, we present a systematic survey of the somatic region of all cells in a cubic millimetre of cortex using quantitative features obtained from electron microscopy. This analysis demonstrates that the perisomatic region is sufficient to identify cell types, including types defined primarily on the basis of their connectivity patterns. We then describe how this classification facilitates cell-type-specific connectivity characterization and locating cells with rare connectivity patterns in the dataset.

## Abstract (web summary)

An analysis demonstrates that quantitative measurements of perisomatic ultrastructure features of neurons can be used to categorize them into cell types.

## Main

**Fig. 1:**
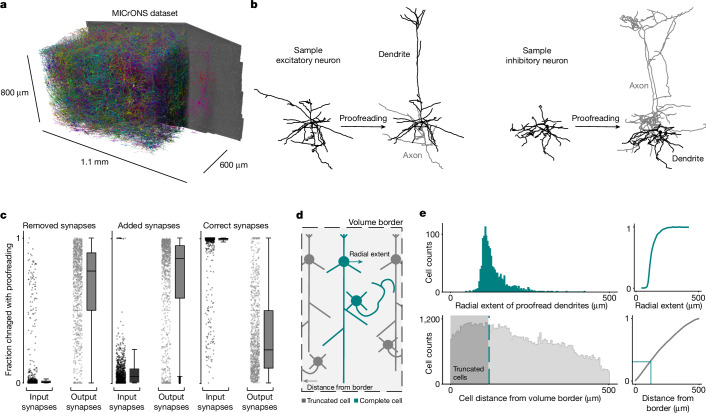
Large-scale automated segmentations necessitate proofreading insensitive cell classifications. **a**, Rendering of a small fraction of neurons from the MICrONS dataset (1.1 mm × 800 μm × 600 μm) covering all layers of cortex and several visual areas, with 1,207 rendered and then cut away to reveal the full morphology of 2 selected neurons on the right portion of the dataset. **b**, Example neuronal morphologies before and after proofreading. Left, excitatory neuron; right, inhibitory neuron. **c**, Fraction of input and output synapses removed (left), added (middle) and maintained (right) after proofreading for 1,347 neurons. For all box plots: centre line, median; box limits, upper and lower quartiles; whiskers, 1.5× interquartile range; outliers not shown (visible in the adjacent scatter plots). **d**, Neurons near the volume borders will have truncated morphologies. **e**, Top: histogram of the radial extent of dendrites from a sample of 1,347 proofread neurons^15^ (left) and the cumulative distribution of those cells (right). Bottom: histogram of the minimum distance from a volume border for all high-quality nuclear detections (*n* = 94,010; left) and the cumulative distribution of those distances (right). The grey shading indicates the portion of cells less than the median radial extent (33% of cells), shown in teal.

Electron microscopy volumes provide a unique perspective on neural circuits by enabling dense tracing of individual axons, dendrites and synaptic connections. Recent progress in data acquisition and dense segmentation has markedly increased the capability to acquire large-scale datasets^5–11^. The size of these volumes allows for large numbers of cells to be analysed with reconstructions of entire dendrites and local axons of mammalian neurons. However, it raises the challenge of accurately classifying tens or hundreds of thousands of cells. Doing so is necessary for many basic investigations, from co-registering neurons, to studying specific cell populations (including neuronal and non-neuronal cells), to characterizing cell-type-specific connectivity at scale. Just as experimental systems require genetic tools to provide inexpensive access to rare cell populations that would otherwise be difficult to study with non-selective techniques, large-scale electron microscopy requires computational tools to provide inexpensive access to specific cell types to facilitate further analyses. Existing methods for automated cell-typing based on morphology or connectivity often necessitate near-complete axonal or dendritic reconstructions^2,12–14^. Such reconstructions require manual correction to the segmentation, often referred to as proofreading, which is prohibitively time-consuming at scale. This means that classifying cells on the basis of specific output connectivity profiles is difficult in the dataset. Moreover, after it is proofread, a single neuron reconstruction contains thousands of pre- and postsynaptic targets to identify (Fig. 1). A method that could identify cell types in the dataset in a way that is insensitive to changes in proofreading and truncation is therefore of high utility, both to automate the classification of targets and to help guide proofreading to cells that have connectivity patterns of interest.

Here we describe a fast, scalable and computationally inexpensive approach that can address these problems. We first analysed the somatic region of nearly 100,000 cells in the MICrONS dataset^5^, a cellular compartment that contains morphological and connectivity-based biological properties that, as we will present, differentiate cell types. By analysing only the somatic region of a cell, our analysis was robust to segmentation errors, unique per cell and thus insensitive to most proofreading changes. We included well-established features known to differentiate cells, such as somatic size and cortical depth, as well as features whose cell-type distinctions are less well recognized such as nuclear folding and soma synapse density. We further developed an unsupervised approach to describe the fine-scale morphology of the perisomatic region of inhibitory cells, and demonstrate that it varies across major inhibitory subclasses. With these features, we address the need for dataset-wide cell-type labels outlined above, by training a hierarchical classifier to identify basic cell classes across the entire dataset. Last, we demonstrate the utility of perisomatic features to facilitate the targeted search for rare cell types across a dataset. This method is already being used to reveal fundamental aspects of cell-type-specific wiring of mammalian cortex^5,15–17^.

## Quality of neuronal arbour segmentation

We analysed the larger segmentation portion of the MICrONS dataset, a 1.1 mm × 800 μm × 600 μm volumetric serial-section electron microscopy dataset from mouse visual cortex^5^, that contains a dense segmentation of cells, a nucleus segmentation and dataset-wide synapse detection^7,18^ (Fig. 1a). This dataset includes 94,010 high-quality nuclear detections enclosed within the boundaries of the volume (Methods) and spans cortical layer 1 to the white matter. For most cells, high-quality cellular segmentation requires proofreading to clean and complete the reconstructions, particularly axons (Fig. 1b,c). Most false mergers are of axonal fragments, leading most outputs of unproofread axons to be incorrect (Fig. 1c). When axonal proofreading is invested in a cell, it creates an elaborate object to analyse with thousands of postsynaptic targets. To analyse the cell-type-specific connectivity pattern of that single reconstructed cell (examples in Figs. 5 and 6), each of those postsynaptic cells requires a cell-type label. Dendrites on the other hand are quite precise, as 75–95% of the 1,000 to 15,000 synapses detected on reconstructed axons can be mapped to their soma in the MICrONS dataset with more than 99% accuracy (Fig. 1c). However, many of these target cells have incomplete reconstructions themselves since, even for volumes of cubic millimetre scale, about a third of the cells are close enough to the edge to have their dendrites truncated (Fig. 1d,e). This level of truncation across cells, whether due to segmentation errors or proximity to the volume border, led us to investigate alternative methods for cell-typing that would be insensitive to a cell’s dendritic and axonal reconstruction status.

## Perisomatic features across cortical cells

**Fig. 2:**
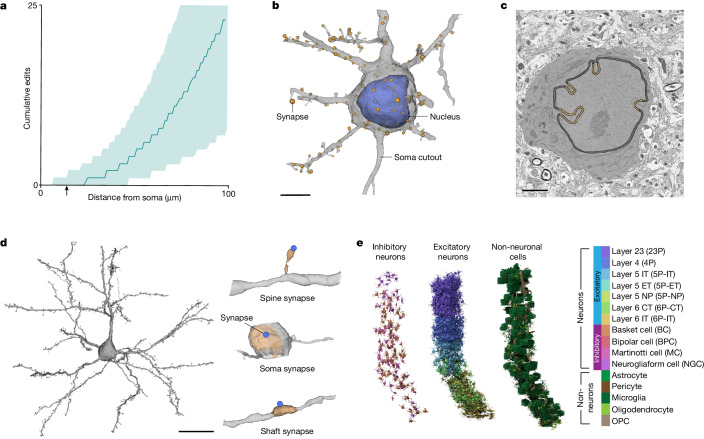
Perisomatic region of cortical cells. **a**, A measure of the distance from the soma for each edit that was made to the segmentation during proofreading of 1,347 cells. The teal line denotes the average and the shading marks the 10th–90th percentile across all cells. The arrow marks 15 µm. **b**, Example cell demonstrating the extent of mesh information used to extract somatic, nuclear and synapse features. All cell meshes were restricted to 15 µm from the centre of the nucleus. **c**, Representative example of nuclear infolding in a single electron microscopy image. The soma is highlighted in grey, black represents the nuclear envelope and orange marks the areas classified as infolded on the basis of the shrink-wrap method (Methods). **d**, Example cell demonstrating the extent of mesh information used to extract postsynaptic features (left) and three example PSSs (right). All synapses included in the PSS analyses were within 60 µm from the centre of the nucleus. **e**, A 3D rendering of the somatic cutouts from all the cells from a 100-µm column that was densely reconstructed for which manual labels were given (*n* = 1,619). Cells rendered are organized by their cell class and coloured by their cell subclass according to the colour scheme displayed. Scale bars, 5 µm (**b**), 2 µm (**c**) and 20 µm (**d**).

The somatic region of the cell is an attractive location for such a method as the automated reconstruction of the somatic region is typically precise and complete (Fig. 2a). Moreover, the soma also has unique biological processes occurring within it, which led us to investigate whether information within the perisomatic region could enable cell classification.

We extracted geometric properties of the nucleus and soma within 15 µm from the centre of the nucleus (Fig. 2b). For nuclei, this included volume, surface area and depth from the pial surface. The three-dimensional (3D) nuclear segmentations provide a detailed view of an notable feature of neuronal nuclei, their tendency to form infoldings of their membranes, sometimes also referred to as invaginations. We quantified the fraction of nucleus membrane area that was within an infolding (Fig. 2c and Methods). Similar geometric properties were calculated for the somatic region including the total volume, surface area, the ratio of the nucleus volume to the soma volume, and the distance from the centroid of the nucleus to the centroid of the soma. We also measured the number and surface density of synaptic inputs detected on the somatic region of the cell. Together these somatic and nucleus features represent a feature space that was extracted for most cells (75% of nuclei detections; Methods). For a subset of neurons, we also analysed the nanoscale structure of the postsynaptic compartments, what we are terming a postsynaptic shape (PSS; Methods) within 60 µm of the nucleus centre (Fig. 2d).

We used a densely reconstructed and manually annotated column of 1,619 cells across all layers of primary visual cortex as the reference dataset for all subsequent analyses^5,15^ (Fig. 2e and Methods). This column included excitatory neurons (1,115), inhibitory neurons (143) and non-neurons (361) with expert-annotated labels for cellular classes and neuronal subclasses (excitatory: layer 2/3; layer 4; layer 5 inter-telencephalic (IT), near-projecting (NP) and extra-telencephalic (ET); and layer 6 IT and cortico-thalamic (CT); inhibitory: Martinotti/non-Martinotti distal targeting cell (MC); basket cell (BC); bipolar cell (BPC); and neurogliaform cell (NGC); non-neurons: astrocyte; oligodendrocyte precursor cell (OPC); oligodendrocyte; microglia; and pericyte; Fig. 2e and Methods). Although we used the above cell-typing scheme throughout our analyses, it should be noted that our approach can incorporate alternative labels as cell-type definitions evolve.

**Fig. 3:**
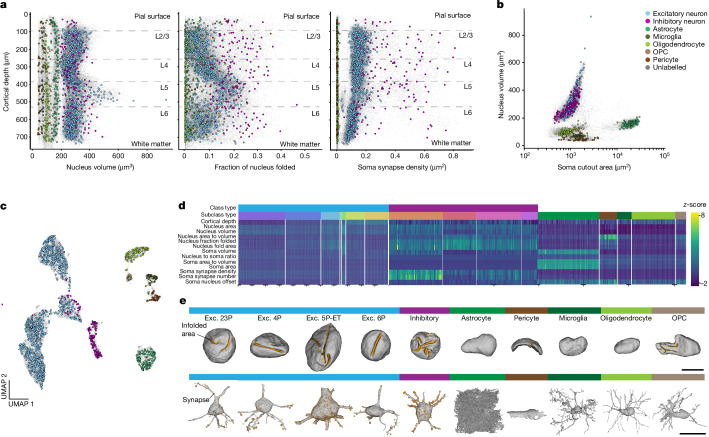
Variations of nucleus and somatic features show stark laminar and cell-class-based distinctions. **a**, Nuclear volume (μm^3^; left), fraction of nuclear membrane infolded (middle) and somatic synapse density (μm^2^; right) against distance from the pial surface (μm). Cortical layer boundaries are marked by dashed lines. **b**, Somatic surface cutout area (μm^2^; within 15 µm from the nuclear centre) against nuclear volume (μm^3^). **c**, 2D UMAP embedding of all neuronal and non-neuronal cells inferred from somatic features, nuclear features and cortical depth. **d**, *z*-scored feature matrix representing all the somatic and nuclear features on the manually labelled cells from the cortical column. Cells are organized by their annotated subclass. Tick marks along the *x* axis denote segments of 100 cells (1,115 excitatory neurons, 143 inhibitory neurons, 361 non-neurons). For all plots, manually labelled cell classes are represented in colour (1,619) and unlabelled examples are shown in light grey (*n* = 92,391). **e**, 3D mesh renderings of representative examples of different neuronal and non-neuronal cell classes. In the top row, nuclei are shown with the folded surface area highlighted in orange. Corresponding cell bodies are shown in the bottom row with somatic synapses in orange. Sphere size corresponds to predicted synapse size from the synapse detection model^5^. Exc., excitatory. Scale bars, 5 μm (top row) and 15 μm (bottom row).

**Extended Data Fig. 1:**
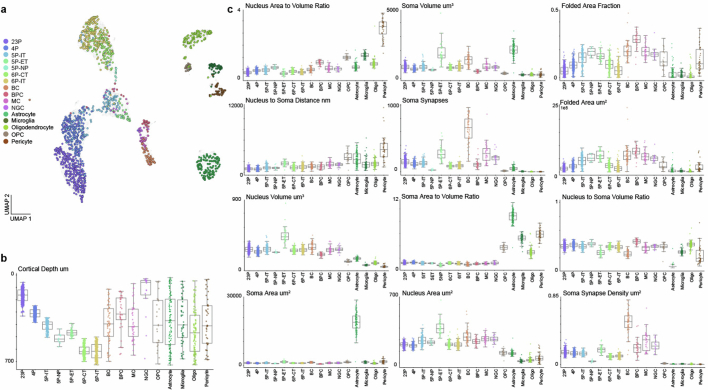
Neuronal and non-neuronal subclass distribution of individual soma and nucleus features. **a**) 2D UMAP embedding of all neuronal and non-neuronal cells inferred from somatic features, nuclear features and cortical depth. Manually labeled cellular subclasses are represented in color (1,619) and unlabeled examples in light gray (n = 92,391). **b**) Distribution and variation of cortical depth of all cells from the manually labeled column dataset. **c**) Distribution and variation of nucleus and somatic features of all cells from the column dataset. For all box plots, center line, median; box limits, upper and lower quartiles; whiskers, 1.5x interquartile range, outliers not shown. Individual cells, including outliers, are shown in the overlaid swarm plots. Color denotes human assigned subclass labels.

To investigate the efficacy of different features in separating cells, we plotted the variability of individual features and trained classifiers to distinguish cells at different levels of granularity. Nucleus features alone were sufficient to separate neurons from non-neuronal cells as non-neuronal cells had smaller nuclei compared to neuronal cells (cross-validated classification accuracy 90%; Extended Data Table 1). Each non-neuronal cell class exhibited a distinct range and consistency in the nucleus volume of its cells (Fig. 3a, left), and thus a nucleus-only classifier was able to identify non-neuronal subclasses with a cross-validated accuracy of 94% (Extended Data Table 1). Nucleus features of excitatory neurons recapitulated expected laminar organization, wherein the borders between layer 2/3 (L2/3), layer 4 (L4), layer 5 (L5) and layer 6 (L6) are all demarcated by shifts in the distribution and variation of nucleus volumes (Fig. 3a, left). Inhibitory cells, on the other hand, had less striking laminar patterns, but had a wider variation of nucleus volumes, overlapping with excitatory cells, with the exception of the larger layer 5 excitatory neurons (Extended Data Fig. 1).

The fraction of membrane inside an infolding also varied depending on depth (Fig. 3a, middle, and Fig. 3e). Neurons in layer 2/3 had smooth nuclear membranes, whereas there was a clear gradient of infolding in layer 4. All layer 5 excitatory cells had high degrees of infolding, despite the notable diversity of cell types and sizes in that population^19^. The degree of infolding decreased again in layer 6 (Fig. 3a, middle). By contrast, inhibitory nuclei had 15–30% of their membrane within an infolding, regardless of their position within cortex. This made them quite distinct from excitatory neurons in layer 2/3, 4 and 6 of cortex, but highly similar to those in layer 5 (Fig. 3a, middle, and Extended Data Fig. 1). Non-neuronal cells generally did not have infoldings, although microglia, OPCs and oligodendrocytes had less spherical and convex shapes (Fig. 3e). Pericytes had the smallest overall volumes with shapes dominated by their close apposition to the vascular walls (Fig. 3e).

Two features alone, nucleus volume and soma cutout area, revealed a striking separation between the main cell classes found in the brain (Fig. 3b). Neurons were separated from all non-neuronal classes and each non-neuronal class occupied distinct portions of this 2D space. The large surface-area measurement for astrocytes was explained by the high density of their processes near the soma. Moreover, the high prevalence of segmentation mergers of pericytes with cortical vasculature resulted in variability in their soma size features as represented by the range in soma cutout area (Fig. 3b). Including the somatic features along with the nucleus features, we trained a classifier to distinguish neurons, non-neurons and erroneous segmentations from each other with a cross-validated accuracy of 95.6%, and a classifier on non-neuronal classes with 97.5% accuracy (Extended Data Table 1).

Excitatory neurons showed a consistent synapse density that varied in a linear fashion with depth through the cortical volume. There was a notable increase in variation in layer 5 that correlated with the three subclasses found there with ET cells having larger synapse densities, NP cells with low synapse densities and IT cells in between (Fig. 3a, right, and Fig. 3e). Inhibitory cells had a much larger density of somatic innervation than excitatory cells, but also have a much wider degree of variation, reflecting previously recorded diversity of inhibitory subclasses^18,20–23^ (Fig. 3a, right, and Fig. 3e). Classifier accuracy for excitatory subclasses was high (90% Extended Data Table 1). Most of the confusion surrounded IT cells located at laminar borders, which corresponds with general areas of disagreement among expert annotators. Notably, accuracy was high across the layer 5 cell types (99% for NP, 85% for IT and 87% for ET).

**Fig. 4:**
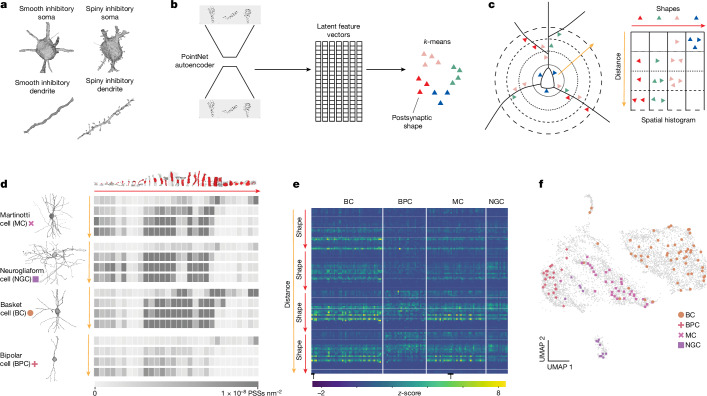
PSS features. **a**, Inhibitory neurons exhibit great variability in ultrastructural morphology. **b**, Procedure for building a PSS dictionary model. The set of shapes is used to train a PointNet autoencoder that learns a latent feature vector of a fixed size (1,024). This autoencoder is then applied to all shapes in the dictionary to generate a set of latent feature vectors. *k*-means with *k* = 30 is applied to this to obtain a set of cluster centres for binning the shapes. **c**, For each cell, the PSSs are binned by shape type and distance from the soma (4 bins) from 0 to 60 µm with 15 µm bin sizes. The resulting histogram is a 2D histogram shown above with the shapes in the *x* direction and distances in the *y* direction. **d**, Examples of 60-µm cutouts of the four predicted inhibitory subclasses with their spatial histograms shown as heat maps. The top row shows the shape of the cluster centre of each of the 30 clusters. In each heat map, darker rectangles indicate higher values. **e**, *z*-scored feature matrix representing the distance-binned PSS features on the manually labelled inhibitory cells from the cortical column (*n* = 143). Cells are organized by their annotated subclass. Tick marks along the *x* axis denote segments of 100 cells. **f**, 2D UMAP of all the inhibitory neurons (*n* = 6,805) inferred after concatenating nucleus, soma and PSS features, with cortical column cells in colour and dataset-wide inhibitory neurons in grey.

To gain a broader understanding of the perisomatic feature landscape, we computed a low-dimensional embedding based on both nucleus and somatic features (Fig. 3c). Consistent with the diversity observed in individual features, the variations observed from the manually labelled cortical column (cell class colors, *n* = 1,619) were reflected in the low-dimensional embeddings of the feature space across all of the cells in the dataset (grey, *n* = 92,391). Non-neuronal cell classes occupied distinct areas of the feature space whereas excitatory neurons were primarily organized by cortical layers (Extended Data Fig. 1). Inhibitory neurons were largely restricted to distinct clusters within this space, with some cells overlapping with cortical layer 5 cells owing to the increase in nuclear infolding in those excitatory neurons. Although there were broad differences in the nucleus and somatic features between the main interneuron subclasses (accuracy of 90%; Fig. 3d and Extended Data Table 1), on inspection, it was clear that the ultrastructure of the perisomatic regions of inhibitory neurons was diverse in ways that the soma and nucleus features did not capture (Fig. 4a).

## Proximal PSSs across inhibitory neurons

Proximal inhibitory branches varied in calibre and surface texture, from smooth and uniform to being covered in small spine-like protrusions (Fig. 4a). To take advantage of this, we developed a method to summarize these fine shape statistics of the proximal arbour^24^. We used the automated synapse detections to identify areas on the dendrites where changes in fine structure (for example, spines) may occur. This approach gave us the added advantage of combining information about synaptic innervation with the fine morphological structure of the postsynaptic neuron surrounding any given synapse.

We computationally segmented the compartment on the postsynaptic side of each synapse, which we refer to as the PSS^24^. This shape, computed as a variable-sized mesh, represented a portion of the soma, the shaft of a dendrite or a spiny protrusion, although it could also be an axon or axon initial segment (AIS). To model the diversity of these shapes, we needed to be able to quantitatively compare them. We therefore trained a PointNet autoencoder that allowed us to generate a fixed-size representation of each shape (Fig. 4b).

**Extended Data Fig. 2:**
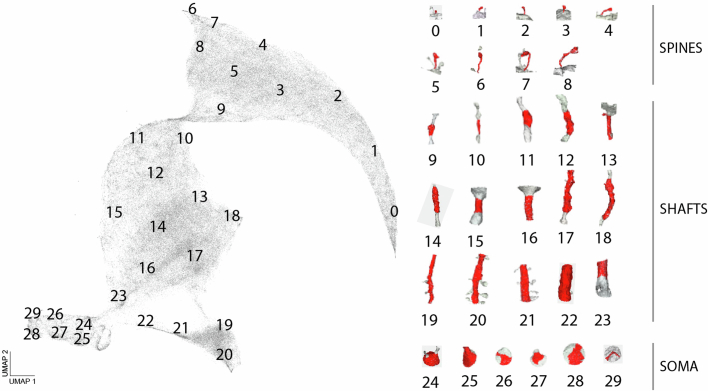
PSS embedding space organized by postsynaptic ultrastructural morphologies. 2D UMAP embedding of all shapes in the PSS Dictionary. The numbers indicate the bin centers mapped in this 2D space and the corresponding PSS meshes on the right show the shape associated with each bin center. Bins 1–8 range in spine shapes, Bins 9–23 are shaft shapes and Bins 24–29 are soma shapes.

To measure the distribution of shapes present in a cell, we collected 236,000 PSSs from a variety of neurons and applied a 2D reduction to visualize their distribution. This resulted in a continuous latent space in which PSS objects of similar morphological character were closer together (Extended Data Fig. 2). We summarized this PSS space into a 30D histogram using *k*-means, to describe the distribution of shapes within a cell (Methods).

**Extended Data Fig. 3:**
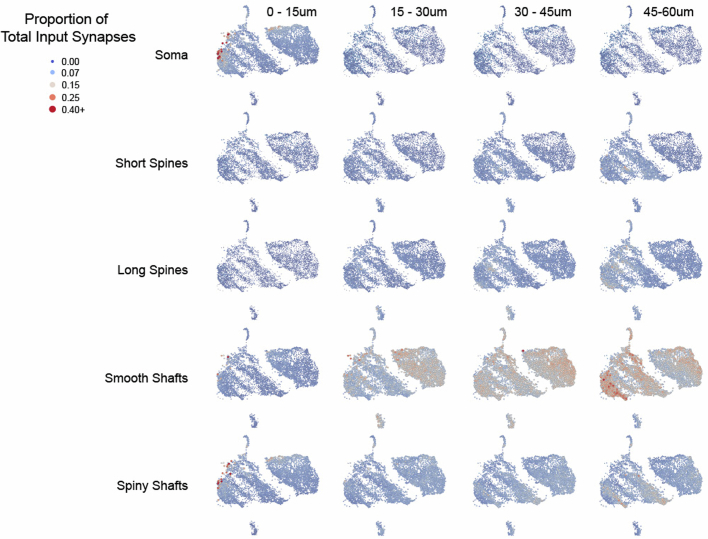
Inhibitory neuron subclasses exhibit spatial patterns to PSS distributions. The UMAP embedding of all the perisomatic features, including PSS features, across all inhibitory cells, colored with respect to what fraction of that cell’s input (within the 60 µm cutout) comes from what PSS/distance bin. PSS shape bins were simplified from 29 bins to 5 broad categories to simplify the visualization (bins 0-4: short spines, 5–8: long-spines, 9-18 + 23: smooth shafts, 19–22: spiny shafts, 24–29: soma). This visualization gives insight into how different cells in different parts of this embedding space receive varying amounts of input onto different shapes within different spatial zones of the perisomatic area. Cells on the far left hand side of the embedding, where in general bipolar type neurons were found, have larger fractions of their inputs near the soma, including dendritic shafts which are more irregular in shape (“spiny shafts”), and smooth shaft inputs farther away where the dendrites begin to elaborate. Basket cells on the right hand side of the side of the embedding are dominated by somatic inputs and smooth shaft inputs which are more evenly distributed spatially. The island at the bottom that is dominated by neurogliaform cells is characterized by having relatively fewer somatic inputs, but an increasing amount of shaft and spiny input at distal dendrites.

We observed that the location of the PSS could further distinguish between cells. For example, although spiny protrusions were most often found on the dendrites of cells, some cells also had them on the soma (Fig. 4a and Extended Data Fig. 3). Therefore, we took a second step to summarize a cell’s distribution of PSSs by adding distance from the nucleus centre. For distance binning, we used four 15-µm bins between 0 and 60 µm from the soma (Methods). Combining the shape and distance binning resulted in a 120D spatial shape histogram (Fig. 4c) that summarizes information about the spatial organization of dendritic shapes and synapse densities near the soma, similar to a multi-dimensional Sholl analysis^25^. There were clear visual differences in the spatial histograms of different cell types (Fig. 4d,e and Extended Data Fig. 3). For example, a Martinotti cell had a greater density of synapses onto small protrusions on its proximal dendrites than the basket or bipolar cell, but similar numbers to the neurogliaform cell. However, the neurogliaform cell had very few synapses on its soma, whereas the Martinotti had many, both onto smaller protrusions and smoother compartments of its somatic compartment (Fig. 4d).

We extracted these features on most putative inhibitory neurons in the dataset (as predicted by perisomatic features; Methods). Appending these features to the soma features increased the accuracy of the inhibitory subclass classifier to 94% (Extended Data Table 1). This was also reflected in the 2D uniform manifold approximation and projection (UMAP) embedding, in which inhibitory subclasses were more separable (Fig. 4f).

## Dataset-wide classification

**Fig. 5:**
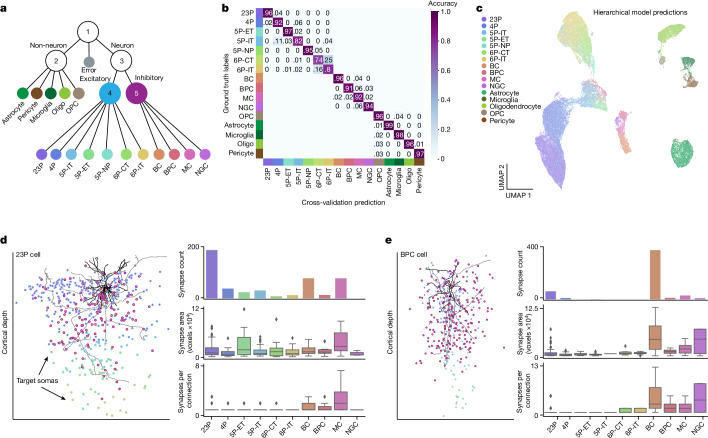
Hierarchical predictions enable dataset-wide circuit analyses. **a**, Diagram of the hierarchical model framework used to predict neuronal and non-neuronal subclasses using a set of five classifiers. Nucleus and soma features alone were used for models 1–4. PSS features were added to predict inhibitory subclasses in model 5 (Extended Data Table 1). Oligo, oligodendrocyte. **b**, Confusion matrix of the cross-validation performance for all cells within the manually labelled column. Note that classifiers for excitatory neurons, inhibitory neurons and non-neurons were trained separately (models 2, 4 and 5 in **a**). The confusion rate between these classes can be seen in Extended Data Fig. 4. **c**, 2D UMAP embedding inferred from depth, nucleus and soma features of all cells in the dataset coloured by the hierarchical model predictions (*n *= 94,010). **d**, Left: 2D rendering of a representative 23P cell morphology, with dendrite in black and axon in grey. Points represent the somatic position of all downstream target cells coloured by the hierarchical model subclass prediction. Right: synapse count (top), total synapse area (middle; voxels are 4 × 4 × 40 nm) and number of synapses per connection (bottom) displayed by the model-predicted subclasses illustrating the local targeting profile of this individual cell. **e**, Similar information as in **d** but for an inhibitory bipolar cell that is predicted to preferentially target basket cells. This unique population of bipolar cells has been further characterized^15^. For all box plots: centre line, median; box limits, upper and lower quartiles; whiskers, 1.5× interquartile range; outliers shown.

**Extended Data Fig. 4:**
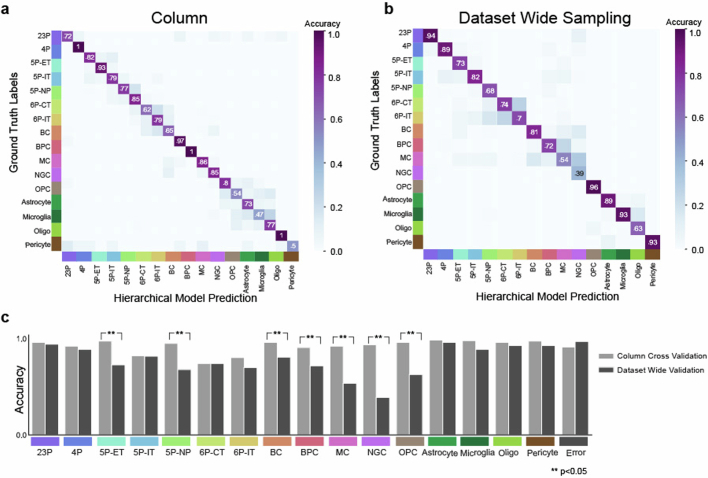
Classifier validation. **a**) Confusion matrix of hierarchical model performance for all cells within the manually labeled column after training. **b**) Confusion matrix of hierarchical model performance on a dataset wide sample of 100 cell predictions from each subclass. **c**) Comparison of column cross validation vs. dataset wide model performance, asterisk notes significance by two-tailed Fisher Exact Test.

**Extended Data Fig. 5:**
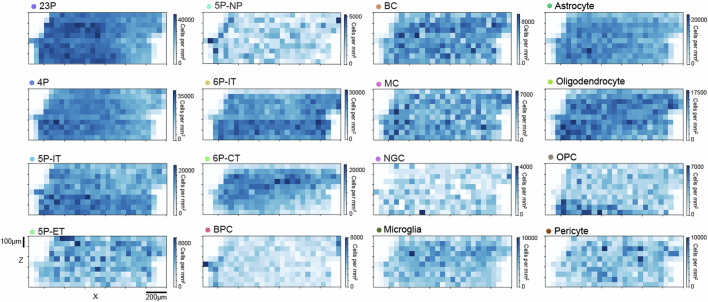
Cell densities across the dataset by cellular subclass. Predicted cell densities per mm^2^ for each subclass across the entire dataset in the XZ plane. Each square represents 50 micron^2^ and color denotes the density scaled per mm^2^. Note due to the approximate 1 mm depth of cortex, these values are also roughly densities per mm^3^. They roughly agree with densities of cells estimated from light microscopy stereology of subclasses^26^, usually utilizing histochemical markers or genetic tools. For some subclasses, there is not a 1-1 to alignment between the definitions of types in this study and the usual molecular markers used in those studies, as molecular markers are not directly measurable in this electron microscopy volume.

**Extended Data Fig. 6:**
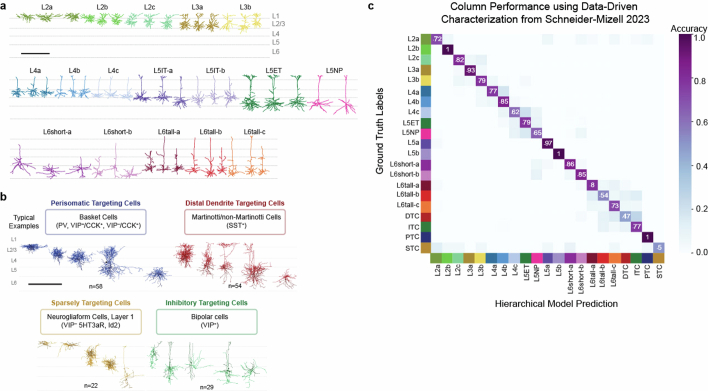
Perisomatic feature based classification utilized with different cell-type labels. **a**) Alternative excitatory subclass labels in the column from Schneider-Mizell et al.^15^, based on unsupervised clustering of dendritic and synaptic features rather than manual human expert calls. Labels on the clusters were inferred based on the overlap with expert labels and cortical depth, with finer distinctions added when necessary (i.e. L4a, L4b, L4c). **b**) Alternative inhibitory subclass labels from Schneider-Mizell et al.^15^ in the column based on unsupervised clustering of their output connectivity statistics. These subclasses (Perisomatic Targeting, Distal Targeting, Sparsely Targeting and Inhibitory Targeting) likely largely but not completely align with broad molecular distinctions made amongst inhibitory cells, based on reviews of the literature where molecular and output connectivity has been measured in the same cells. **c**) A confusion matrix of a hierarchical model retrained to utilize these subclass labels for excitatory neurons vs inhibitory neurons rather than human expert labels. Cross validation performance on the excitatory (67%) and inhibitory (85%) subclass models was lower than the expert labels, due primarily to the fine grained distinctions made amongst layer 4 and 6 types. The confusion matrix shown here is the output of the final model trained on all samples from the column.

To enable dataset-wide classification, we developed a hierarchical model that used a cascade of classifiers to sort cells at increasingly finer distinctions. Classifiers were integrated into a comprehensive model in which individual cells are sorted down the hierarchical tree (Fig. 5a). We used a collection of classification models (support vector machines or multilayer perceptrons) on different feature sets (nucleus alone; nucleus and soma; nucleus, soma and PSS) and found an optimal combination of classifiers that predicted cell types labelled within the column with an overall accuracy of 91% (Fig. 5b and Extended Data Table 1). All classifiers were trained using the labels from the manually labelled cortical column (see Methods for more detail). Notably, this provided classifications for 88% of the cellular objects in the dataset (94,010/106,761 cells). To further validate this classification, we randomly sampled 100 cells from each subclass predicted by the hierarchical model and had anatomical experts assess the labels (Extended Data Fig. 4). For many classes, the average classification in this validation was consistent with performance accuracy within the column. The lower validation accuracy in the inhibitory subclasses as well as 5P-ET and 5P-NP was related to the sparse sample sizes in the training data from the column. The largest single confusion between types here was between adjacent layers of similar pyramidal classes, for which strict laminar boundaries separating manual classes are less confident. This demonstrates that these features are indeed useful for separating cell types on the basis of local somatic reconstructions of cortical cells, consistent with the structure of the low-dimensional embedding (Fig. 5c). Furthermore, predictions of cell density and overall cell counts per subclass across the dataset (Extended Data Fig. 5) corroborate the sampling rates we would expect from previous studies^26–29^. This approach can be adapted to accommodate new cell-type labels derived from more detailed or expansive studies of the dataset, creating a scalable platform for extending labels derived on smaller numbers of cells to dataset-wide coverage. For example, we have trained models on the basis of the unsupervised clustering labels of morphological and connectivity properties of the same column cells as described previously^15^ (Extended Data Fig. 6).

Dataset-wide classifications enable a range of subsequent analyses. The typical axon of a well-proofread neuron has hundreds or thousands of postsynaptic targets^5^. To quantify the cell-type-specific connectivity of such cells, each of those targets should have a cell-type label. Doing so manually is a practical bottleneck in analysing these data. With these predictions, scientists can analyse the most numerous postsynaptic targets, the weight of these synapses with respect to predicted synapse area, and the number of synapses between proofread cells and cell subclasses across thousands of synapses (Fig. 5d,e). For example, a given layer 2/3 pyramidal neuron made the most synapses onto other 23P neurons (Fig. 5d). However, when we looked at the total predicted synapse size, 5P-ET neurons receive some of the largest average synapses. Some examples were more unexpected than that of the 23P cell. For example, bipolar cells (which largely overlap with a VIP subclass) have been described as the only disinhibitory specialist interneuron class, and are described as making synapses primarily onto SST (somatostatin) cells (which are thought to overlap with the Martinotti cell definition used here)^30–33^. Although the dataset contains cells consistent with that view, a companion study on extensively proofread cells identified a collection of disinhibitory multipolar neurons that exhibit strong targeting preferences for basket cells^15^. This unique connectivity profile is observed in the dataset-wide classifications as well (Fig. 5e).

## Efficient search for rare cells

Studying the connectivity patterns of cell types requires identifying many example cells of a particular connectivity profile. With more than 70,000 neurons sampled across a millimetre scale there should be many examples of any individual cell type. However, locating those examples can be challenging for rare subclasses because of their infrequent appearance and the need for axonal proofreading to use connectivity to suggest their subclass.

**Fig. 6:**
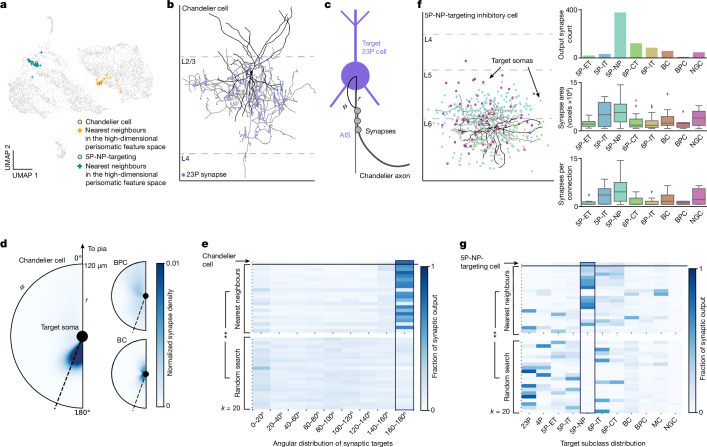
Perisomatic feature space enables more efficient search for unique cells. **a**, 2D UMAP embedding highlighting a chandelier cell (orange dot), a 5P-NP-targeting cell (blue dot) and their respective 20 nearest neighbours in the high-dimensional perisomatic feature space. Note, UMAP nonlinearly distorts feature space, so not all nearest neighbours appear closest in the plot. **b**, Example proofread chandelier cell in L2/3 (dendrite in black, axon in grey). Output synapses are marked along the axon and coloured by subclass prediction. Note the characteristic vertical chains of synapses onto 23P cells. **c**, Chandelier cells are characterized by their preference to synapse onto the AIS of target cells^34,35^ (quantified here by measuring the angle between the target soma and the synapse (*ϕ*) and the distance from the soma (*r*)). **d**, Heat map illustrating the angle and distance distribution of the chandelier cell shown in **b** as well as two non-Chandelier inhibitory examples. Colour denotes the normalized synapse density for each cell. Synapses located at an angle >160° were considered onto the AIS of the target cell (shown by the dashed line). **e**, Angular distribution histogram of the chandelier cell (top row), 20 nearest neighbours in the perisomatic feature space, and 20 random inhibitory cells (*P* < 0.00001). **f**, Example cell that preferentially targets the rare 5P-NP subclass (dendrite in black, axon in grey); points represent target cell soma locations coloured by predicted subclass. Output synapse counts reflect strong preference to 5P-NP cells. For all box plots: centre line, median; box limits, upper and lower quartiles; whiskers, 1.5× interquartile range; outliers shown. **g**, Fraction of output connectivity onto neuronal subclasses of the 5P-NP-targeting cell (top row), 20 nearest neighbours in the perisomatic feature space, and 20 random inhibitory cells (*P* < 0.00001). All *P* values reported and asterisks represent significance by two-tailed Fisher exact test.

Given that the main inhibitory neuron subclasses differ in their connectivity profiles, we already had some evidence that connectivity profile correlates with the perisomatic features we extracted (Fig. 4e), but we conjectured that they could be useful for finding rarer types with specific connectivity patterns for which we did not yet have labels. One well-known rare cell type in mouse visual cortex is the chandelier cell, which synapses onto the AIS of excitatory neurons^20,34–38^. We used a single proofread chandelier cell to determine whether we could facilitate finding other cells like it using the perisomatic features. We selected the top 20 nearest neighbours of the perisomatic feature space (Fig. 6a) and assessed what fraction of them were chandelier cells on the basis of their connectivity profiles after cleaning them of false mergers and modest axonal extension (Methods). The chandelier cell’s connectivity profile is easy to recognize, both from its morphology where it makes vertical strings of synapses (Fig. 6b), and the unique targeting of synapses onto the AIS of excitatory neurons. As the AIS is located just below the soma of excitatory cells in the cortex, the angular distribution of synapses relative to somatic targets can be used as a spatial proxy for AIS-targeting (Fig. 6c,d). A histogram of the angular distribution of synapses relative to the target soma demonstrates that 16 of the 20 nearest-neighbour cells have connectivities consistent with chandelier cells (Fig. 6d,e). By contrast, none of the 20 random interneurons we sampled from the inhibitory neurons in the dataset, or any of the 143 interneurons sampled in the column, was a chandelier cell, reflecting a significant enrichment (*P* < 0.00001 by two-tailed Fisher exact test). On the basis of this success, we tried to find more examples of cells with a less well-known connectivity profile. We selected an undescribed but proofread interneuron that made most of its synapses in layer 5 onto 5P-NP neurons, despite those pyramidal neurons themselves being rare and with few input synapses^39^ (Fig. 6f). Picking the top 20 nearest neighbours of this cell, we found 13 cells that made at least 30% of their synaptic targets onto 5P-NP cells (on the basis of classifier predictions). This stands in contrast to the case of 0 of the 20 random interneurons we sampled, or 2 cells of the other 143 sampled in the column, again a significant enrichment (*P* < 0.00001; Fig. 6g). This application demonstrates how these perisomatic features can facilitate the search for rare cell types in the cortex.

## Discussion

Our analysis of the perisomatic region of cells in the mouse visual cortex demonstrates that a substantial amount of cell-type information can be extracted from the somatic regions of brain cells. Our approach has already been used to characterize the connectivity of distinct types of layer 5 Martinotti cell^16^, the inter-related connectivity motifs of layer 5 thick tufted cells (5P-ET) and the surrounding inhibitory subnetwork^17^, and to confirm the connectivity profiles of interneurons outside the manually labelled column^15^. Future work in this dataset and others will leverage iterations of dataset-wide cell classifiers to discover new aspects of cell-type-specific wiring of cortical circuits. Other cell classification approaches have been applied to this dataset, including unsupervised clustering of morphological features, and supervised approaches based on morphological graphs^40,41^. All of these approaches have focused on smaller subsets of the data that contained higher-quality or complete reconstructions, reducing their effective coverage in the datasets to less than half the cells.

The breadth of cells in large-scale electron microscopy data makes it an attractive modality to study cell types. Our approach provides an example of how computational methods are an important practical tool for directing study to small subsets of cells within large datasets. This is particularly apparent for diverse and rare inhibitory cells (Figs. 5 and 6); however, this approach can be applied to other diverse cell classes such as glia. One such example is the difference between OPCs and premyelinating oligodendrocyte cells, which are thought to be differentiated OPCs that are in transitional states to oligodendrocytes^42^. The structural diversity of cells predicted as OPCs within the low-dimensional embedding space (Fig. 5c) suggests that searching within the perisomatic feature space, as illustrated in Fig. 6, could be used to facilitate further scientific discovery across brain cell types. More broadly, some of the features described here can be measured with other techniques, such as X-ray tomography or light microscopy, and can be used to separate cells into different subclasses in a manner similar to what has been presented here.

Many studies of anatomical diversity of cortical cells have focused on the diversity of dendritic and somatic morphologies, axonal projection patterns and synaptic connectivity patterns^3,4,21,43^. Fewer studies have focused on differences of somas^44–46^; in particular, there have been few quantitative studies of the 3D ultrastructure of the soma with large single-cell sample sizes across all layers of cortex. Laminar differences in cell body size distributions are well known, and serve as the basis for cyto-architectural definitions of layers, which corresponds with cell-type-specific shifts in size, specifically excitatory ones^47^. For example, pyramidal layer 5 ET projection neurons are characterized by their large somas. This probably reflects differing demands for gene expression and metabolic load^1^. Also, 5P-NP neurons have been recognized before as having smaller rounder somas on average^19,39^. Further, anecdotal descriptions of variations in nucleus infolding have been reported, although only in two dimensions within a narrower range of types^48,49^. Notably, differences in nucleus infoldings have been reported to be modulated by activity in some brain areas^50^. The results we present here not only corroborate the variability of these features across cells but indicate that the perisomatic region may hold greater cell-type-specific information than previously recognized.

**Extended Data Fig. 7:**
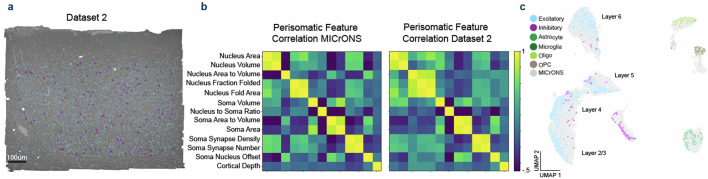
Basic perisomatic feature patterns maintained across a second dataset from a different animal. **a**) A cutout of a second dataset, which covers layer 2/3 to 6 of cortex, but is only 50 µm thick. Somas contained within this volume (n = 1,944) were analyzed in a manner identical to the larger dataset and soma, nucleus and PSS features were extracted. Excitatory nuclei highlighted in light blue and inhibitory nuclei in magenta. **b**) Feature to feature Pearson correlations exhibit similar correlation structure between the two datasets. **c**) A joint UMAP of the perisomatic features with the MICrONS dataset data shown in gray, and the smaller dataset covered by manually identified cell classes overlaid. In general, the same overall patterns and degree of separation amongst layers and cell classes was observed. Note: pericytes were manually excluded from this dataset due to the lower quality of nucleus and somatic segmentations. Extensive detailed subclass cell type validation is not possible in this dataset due to the truncation of axons and dendrites.

There are a few limitations to this work that should be kept in mind when interpreting its results. Our most detailed analysis has been completed on only one dataset that comes from a single animal. That said, some patterns are consistent with what was found in a smaller published dataset from layer 2/3^18,20,51^, and the basic patterns found in these features across mouse visual cortex are reproduced in a second smaller dataset (Extended Data Fig. 7). Our approach is not the final word in cell-type predictions in this dataset, or large-scale electron microscopy in general, and there are a number of directions for potential improvement. Further, cell-type labels will continue to evolve as more cells are classified by either human experts or quantitative methods with increasing specificity and sophistication. In particular, our validation results are consistent with there being a larger diversity of inhibitory cells than exist within the column; thus, expanding the number of class labels could improve performance. However, the dataset-wide framework we have presented here should continue to be valuable, as we expect any new labels to be available for only a subset of cells. Finally, this model does not use all of the information present at the soma of neurons. For example, the detailed ultrastructure visible in the imagery is not fully utilized. Other methods have utilized the underlying imagery of cells to distinguish cell types, either through detection of more subcellular organelles such as cilia or by using imagery more directly to define abstract embeddings^52–54^.

Outside the somatic region, there are a large variety of studies have shown how local features visible in the ultrastructure contain information about cell types, including neurotransmitters of fly synapses, identity of neuromodulatory axons, or cutouts of local dendrite and axons^52,55^. These results all support the view that large-scale quantitative measurements of ultrastructure provide a rich basis for identifying cellular properties of cells. The efficacy of these approaches provides a roadmap for how to develop a scalable platform for leveraging local features of cells to infer cell-type classifications. Beyond neuroscience, this approach illustrates how large-scale ultrastructural imaging of cells can facilitate the study of diverse and rare cell populations if paired with appropriate quantitative analysis.

## Methods

### MICrONS dataset

This dataset consists of a 1.1 mm × 800 µm × 600 µm segmentation of a volumetric serial-section electron microscopy dataset from mouse visual cortex of a male postnatal day 87 (P87) mouse. The dataset covers all layers of cortex and spans primary visual cortex and two higher visual areas. The dataset has been described in detail elsewhere^5^. Briefly, two-photon imaging was carried out on the mouse, which was subsequently prepared for electron microscopy. The specimen was then sectioned and imaged using transmission electron microscopy^6^. The images were then stitched, aligned and processed through a deep learning segmentation algorithm, followed by manual proofreading^5–7,15^.

### Cortical column

In this manuscript, we leveraged proofreading that was carried out and labels that were prepared as part of a separate study of a 100-µm columnar region of primary visual cortex within the larger dataset^15^. For clarity and completeness, we repeat some aspects of the methods that define that column here.

#### Column selection

The column borders were found by manually identifying a region in the primary visual cortex that was as far as possible from both the volume boundaries and the boundaries with higher-order visual areas. A 100 × 100 µm box was placed on layer 2/3 and was extended along the *y* axis of the dataset. While analysing the data, it was observed that deep-layer neurons had apical dendrites that were not oriented along the most direct pia-to-white-matter direction, and thus we adapted the definition of the column to accommodate these curved neuronal streamlines. Using a collection of layer 5 ET cells, points were placed along the apical dendrite to the cell body and then along the primary descending axon towards the white matter. The slant angle was computed as two piecewise linear segments, one along the cortical depth to lower layer 5 where little slant was observed, and one along the direction defined by the vector-averaged direction of the labelled axons.

Using these boundaries and nucleus centroids^5^, all cells were identified inside the columnar volume. Coarse cell classes (excitatory, inhibitory and non-neuronal) were assigned on the basis of brief manual examination and rechecked by subsequent proofreading and confusion analysis with early versions of the classifiers described here. To facilitate concurrent analysis and proofreading, all false merges that connected any column neurons to other cells (as defined by detected nuclei) were split.

#### Proofreading

Proofreading was carried out by five expert neuroanatomists using the Connectome Annotation Versioning Engine^56,57^ and a modified version of Neuroglancer^58^. Proofreading was aided by on-demand highlighting of branch points and tips on user-defined regions of a neuron based on rapid skeletonization (https://github.com/AllenInstitute/Guidebook). This approach quickly directed proofreader attention to potential false merges and locations for extension, as well as allowing a clear record of regions of an arbour that had been evaluated.

For dendrites, all branch points were checked for correctness and all tips were examined to determine whether they could be extended.

False merges of simple axon fragments onto dendrites were often not corrected in the raw data, as they could be computationally filtered for analysis after skeletonization (see below). Detached spine heads were not comprehensively proofread, and previous estimates place the rate of detachment at approximately 10–15%.

For inhibitory axons, axons were ‘cleaned’ of false merges by looking at all branch points. Axonal tips were extended until either their biological completion or data ambiguity, particularly emphasizing all thick branches or tips that were well suited to project to new laminar regions. For axons with many thousands of synaptic outputs, some but not all tips were followed to completion once main branches were cleaned and established. For smaller neurons, particularly those with bipolar or multipolar morphology, most tips were extended to the point of completion or ambiguity. Axon proofreading time differed substantially by cell type, not only because of differential total axon length, but also because of axon thickness differences that resulted in differential quality of auto segmentations, with thicker axons being of higher initial quality. Typically, inhibitory axon cleaning and extension took 3–10 h per neuron. Expert neuroanatomists further labelled excitatory and inhibitory neurons into subclasses. Layer definitions were based on considerations of cell body density (in analogy with nuclear staining) supplemented by identifying kinks in the depth distribution of nucleus size near expected layer boundaries.

#### Cell labelling

For excitatory neurons, the categories used were: layer 2/3 IT, layer 4 IT, layer 5 IT, layer 5 ET, layer 5 NP, layer 6 IT and layer 6 CT cells. Layer 2/3 and upper layer 4 cells were defined on the basis of dendritic morphology and cell body depth. Layer 5 cells were similarly defined by cell body depth, with projection subclasses distinguished by dendritic morphology following ref. ^2^ and classical descriptions of thick (ET) and thin-tufted (IT) cells. Layer 5 ET cells had thick apical dendrites, large cell bodies, numerous spines and a pronounced apical tuft, and deeper ET cells had many oblique dendrites. Layer 5 IT cells had more slender apical dendrites and smaller tufts, fewer spines and fewer dendritic branches overall. Layer 5 NP cells corresponded to the ‘spiny 10’ subclass described in ref. ^2^; these cells had few basal dendritic branches, each very long and with few spines or intermediate branch points. Layer 6 neurons were defined by cell body depth, but only some cells were able to be labelled as IT or CT by human experts. Layer 6 pyramidal cells with stellate dendritic morphology, inverted apical dendrites or wide dendritic arbours were classified as IT cells. Layer 6 pyramidal cells with small and narrow basal dendrites, an apical dendrite ascending to layer 4 or layer 1, and a myelinated primary axon projecting into white matter were labelled as CT cells.

Basket cells were recognized as cells that made more than 20% of their synaptic inputs onto the soma or proximal dendrites of cells. Neurogliaform cells were recognized by having a low density of output synapses, and boutons that often had synaptic vesicles but no postsynaptic structures. Bipolar cells were labelled by having only 2 or 3 primary dendrites, and primarily making synapses with other inhibitory neurons. Note that the Martinotti/non-Martinotti subclass label was given to cells that have previously been described in the literature to primarily target the distal dendrites of excitatory neurons without exhibiting hallmark features of bipolar or neurogliaform cells.

Owing to high levels of proofreading in the column, there were very few errors; thus, the training set was augmented with manually labelled errors from the entire dataset.

### Proofreading and truncation analysis

For every proofread cell in the cortical column (described above), we compared the cellular volume of the initial reconstruction from the automated segmentation to the cleaned and completed reconstruction. To measure the precision connectivity for each cell, we noted the number of synapses that got removed with proofreading, the number of synapses that were added, and the number of synapses that were maintained with each cell before and after proofreading.

To estimate the likelihood of truncation, we measured the distribution of dendritic extents from the proofread column cells. For each cell, we measured the radial distance of each input synapse from the cell’s soma. For each cell, the distance from the soma of every input synapse was calculated and the radial extent was defined as the 97th percentile of this distribution. From a distribution of these measurements across all cells, we used the median value of 121 µm as a threshold for dendritic truncation, although closer to 250 µm would be required to guarantee no truncation for any cell. For the rest of the cells in the dataset, we measured the distance of the soma from the volume borders in *x* and *z*. The overlap in these distributions relates to the probability of truncation, leading to our conclusion that roughly one-third of the cells have some degree of dendritic truncation.

### Generating nucleus and soma features

We analysed nuclei using the results of a deep neural network segmentation^5^, extracted the mesh using marching cubes and obtained the largest component of the detected mesh. Nuclear features were then extracted on the remaining meshes. These features included nucleus volume, nucleus area, the area-to-volume ratio, nucleus surface area within an infolding, the fraction of the total surface area within an infolding, and cortical depth (measured as the distance from the pial surface). Nucleus fold features were extracted by creating a shrink-wrapped^47^ mesh for each nucleus mesh. We then calculated the distance of each vertex on the nucleus mesh from the shrink-wrapped mesh. After visual inspection of cells across all of the reported subclasses, any vertex further than 150 nm was considered to be within an infolding.

For each nucleus detection, the somatic compartment was identified as the ID in the segmentation that surrounded >80% of the nucleus. Somatic segmentations (downloaded at 64 × 64 × 40 nm resolution) went through a heuristic cleaning procedure to remove missing slices of data and incorrectly merged fragments. As each soma was matched to its corresponding nucleus, 15 µm surrounding the nucleus’s centre of mass was cut out from the dense segmentation and converted into a binary mask. The value of 15 µm was chosen owing to the high quality of the segmentation (Fig. 2a) and it was large enough to encompass the entire soma of all cells from the smallest glial cell to the largest 5P-ET neuron. Binary dilation by five voxels in three dimensions was carried out, followed by filling of all holes, and then binary erosion of three voxels. The resulting binary mask was meshed using marching cubes and connected component analysis was run on the result. The value of five voxels was deemed an appropriate dilation to remove merged fragments without creating additional holes in the mesh. The largest connected component mesh was retained, and any disconnected components were dropped. Somatic features were extracted for all nuclear detections that were not cut off by the volume boundary (see the section entitled Filtering procedure). These somatic features included soma area, soma volume, the area-to-volume ratio, the number of synapses on the somatic cutout and the soma synapse density. Using both the somatic and nucleus meshes, we calculated the ratio between the nucleus volume and soma volume and the offset between the two, measured as the Euclidean distance between the nuclear centre of mass and the soma centre of mass.

#### Filtering procedure

There were 133,580 nuclear detections in the dataset, and the filtering procedure consisted of three steps. First, any detected objects less than 25 µm^3^ were filtered out as errors as these largely consisted of small fragments of nucleoli. Second, after identifying the segment IDs within a 15-μm bounding box around each nucleus, if more than 20% of these IDs corresponded to error ID 0, they were filtered out. Most of these error cases were cells close to the volume border or areas in the volume with higher segmentation errors such as those near blood vessels. Third, cells that were predicted as errors by the object classifier of the hierarchical model described below (model 1 in Fig. 5a) were also removed from analysis. This resulted in a final set of 94,010 cells, neuronal and non-neuronal.

#### Feature normalization

Owing to differences in section thickness during sample preparation, we noticed abrupt shifts in nucleus and soma size features along the sectioning axis (*z* plane). This presumably is due to changes in section thickness across the dataset. To account for these abrupt and systematic shifts, we binned the entire dataset by the longest length scale for which there did not seem to be systematic shifts in the distribution in the *z* plane (800 nm) and normalized each feature value by the average within each *z* bin.

For 2D UMAP embeddings and training of the classifiers, it was important to place all features in approximately similar scales. For this reason, we independently *z*-scored each feature across all cells and used that as the input for classifier training as well as the UMAP embeddings in Figs. 3–6.

### Generating PSS features

Around each synapse, we extracted a 3,500-nm region to obtain the synapse region mesh. We experimented with region cutouts between 1,000 and 5,000 nm; however, smaller cutouts led to ambiguities in the main shaft identification and thereby produced errors in the subsequent skeletonization. At 3,500 nm, the skeletons were more stable and segmented as expected. This mesh was then segmented using the CGAL surface segmentation algorithm^59^, which splits regions on the basis of differences in thickness. We adapted our previously developed method^24^ to identify the PSS region by using a local skeleton calculated from the synapse region mesh, rather than a precomputed whole-cell mesh. This allowed us to adapt this method for cells in the dataset without the need for proofreading.

Given a cell for which all PSSs have been extracted within a 60 µm radius from the nucleus centre, the objective was to build a descriptor that encapsulates the various properties of the PSS. In particular, we aim to capture two of these properties: the type of shape of the PSS and the distance of the PSS from the soma. Moreover, as different cells can have different numbers of shapes (synapses), we needed a fixed-size representation for each cell. To capture shape information, a dictionary of all shape types was built using a dictionary dataset from 236,000 PSSs from a variety of neurons^24^. These shapes were rotationally normalized and used to train a PointNet autoencoder^60,61^ to learn a latent representation of size 1,024. The high-dimensional latent space spanning all of these shapes is a continuous space (Extended Data Fig. 3), which was used to generate a bag of words model^30^ for the shapes. To ensure that we were sampling the entire embedding space, we carried out *k*-means clustering with *k* = 30 to estimate cluster centres. We manually reordered the bin centres for visualization purposes from shapes representing small spines, to those representing longer spines, to dendritic shafts of different shapes, and finally somatic compartments. The top row of the right panel of Fig. 4d shows the shape in the dictionary that is closest to each of these cluster centres. For distance from the soma, we split the 60 µm radius around the nucleus centre into four 15-µm radial bins (Fig. 4c,d). All PSSs were then binned according to their shape and distance properties to generate a histogram of counts. Initially we extracted PSSs from within 120 µm radius. However, on inspection of the normalized histograms and the 2D UMAP embedding space, the additional radial bins did not increase our differentiability and did increase truncation effects near the dataset; thus, we reduced the radius to 60 µm. Finally, this histogram was *z*-scored and then added to the rest of the features as input to classifiers and the UMAP embedding (Figs. 4 and 6).

### Hierarchical model training and validation

#### Hierarchical framework

We defined an object as the segmentation associated with a predicted nucleus^5^ from which nucleus, soma and PSS features could be extracted. A hierarchical framework was designed to predict the cell type of any such object (Fig. 5c). To begin, there were 106,761 nuclear segmentations that passed the first 2 filters described above (see the section entitled Filtering procedure). The first level in the hierarchy predicted whether an object was a neuron (72,158), non-neuron (21,856) or an error (12,751). All objects predicted as errors were excluded from all subsequent analyses except for the hierarchical model evaluation. Non-neuronal cells were then classified as one of the following: astrocyte (7,850), microglia (2,638), oligodendrocyte (7,020), oligodendrocyte precursor cell (OPC; 1,703) or pericyte (2,645). For neurons, cells were predicted as either excitatory (64,195) or inhibitory (7,963) followed by a separate subclass classifier for each class type. Excitatory subclasses were layer 2/3 pyramidal (19,735), layer 4 pyramidal (14,777), layer 5 IT (7,949), layer 5 ET (2,215), layer 5 near-projecting (NP) pyramidal (970), layer 6 IT (11,734) and layer 6 CT pyramidal (6,815). After extracting PSS features from all predicted inhibitory neurons, a subset of neurons (*n* = 1,158) that were actually excitatory clearly separated from the rest of the cells in the perisomatic feature space (with PSS features). This was expected owing to known differences in proximal dendrite morphology between inhibitory and excitatory neurons. These neurons were then passed through the excitatory neuron classifier and labelled as excitatory for all subsequent analyses with a final set of 6,805 inhibitory cells with the following subclass counts: basket cells (3,239), bipolar cells (997), Martinotti/non-Martinotti cells (1,992) and neurogliaform cells (571).

#### Training

Soma and nucleus features were extracted from the 3D mesh of all objects and PSS features were extracted for all neurons predicted as inhibitory. For each level of the hierarchy, multiple classifiers were trained using either nucleus alone, nucleus and soma features, or nucleus, soma and PSS features. Within each level of the hierarchy, classifiers were trained using the cells and labels from the manually annotated cortical column. Owing to the sparsity of some of the cell classes, we augmented the training set in the following ways: 470 errors were added from within and around the column for the object model; 11 proofread 5P-NP cells and 250 proofread 5P-ET cells were added to train the excitatory subclass model.

For each classifier, the model type was chosen using a randomized grid search for the following models: support vector machine with a linear kernel, support vector machine with a radial basis function kernel, nearest neighbours, random forest classifier, decision tree and neural network. For each type, 50 models were trained with varying parameters and the top-performing model was chosen. Individual models were further optimized using tenfold cross-validation evaluated on the basis of accuracy and F1 score (a measure for precision and recall). Training and test examples were held consistent across models for direct performance comparison within each level.

#### Model performance and validation

The hierarchical model was defined as the sequential combination of the best-performing classifiers at each level. To see the performance of all different feature sets at each level of the hierarchy, see Extended Data Table 1. The overall performance of the hierarchical model was measured with a test set that involved manual inspection of 100 examples of each of the neuronal and non-neuronal subclasses as well as errors. This resulted in a test set of 1,700 cells. Cross-validation and test performance for the hierarchical model are reported below (Extended Data Fig. 4). Note that all scores reported are the weighted accuracy based on the sampling rate of each class within the column.

The top level of the hierarchy (the object model), distinguished neurons from non-neurons as well as erroneous detections. The cross-validated accuracy score on the column was 96% with a test score of 97%. The second level of the model simply distinguished excitatory from inhibitory neurons. Here, the column cross-validated accuracy score was 94% and the test set was 93%. Overall, across all subclasses, the hierarchical model on the column had a cross-validated accuracy of 91% and a dataset-wide test set accuracy of 82%.

### Chandelier cell identification

Chandelier cells are characterized by their unique axo-axonal synapses onto the AIS of target pyramidal cells. As there were no chandelier cells within the densely reconstructed column, we sought to test whether the perisomatic feature space would facilitate an enriched dataset-wide search for these cells. After identifying and proofreading a chandelier cell, we selected the top 20 nearest neighbours by Euclidean distance using a KDTree search of the perisomatic feature space (nucleus, soma and PSS features) after *z*-score normalization of each feature across cells. We also selected 20 random cells from the predicted inhibitory neurons. For each of these 40 cells, we proofread the reconstructions to ensure that there were no extraneous neurites attached, and extended the axon until there were at least 100 output synapses. On average, each of the 20 nearest neighbours had 590 output synapses attached and the random cells had 809 synapses attached.

To quantify whether a given cell was a chandelier or not, we measured the angle (*ϕ*) and the distance (*r*) between every output synapse and the soma of the postsynaptic cell (Fig. 6c). A synapse with an angle value of 0° would be considered to be directly above the target soma whereas one with an angle of 180° would be considered to be below it. Owing to variations in axon directionality with respect to the pial surface, we considered synapses with angle values between 160° and 180° and within 60 µm of the soma to be on the AIS of the target soma. In fact, because the specificity of chandelier targeting is so high, the density of synapse angle distributions alone was enough to identify other chandelier cells (Fig. 6e). On inspection of the proofread 20 nearest neighbours, we determined that cells with more than 40% of their synapses within 160–180° were chandelier cells. The average normalized density for the identified cells was 62% as compared to 8% for the non-chandelier cells. A two-tailed Fisher exact test was carried out to test significance between the random cell population and the nearest neighbours.

### Inhibitory neuron output targeting

After characterizing a single 5P-NP-targeting cell, we applied a similar strategy to the one above to search for more neurons in the dataset that had a similar connectivity pattern. We selected the top 20 nearest neighbours by Euclidean distance in the perisomatic feature space using a KDTree search. These cells were proofread to remove false mergers and the axon was extended to include at least 100 synapses. It should be noted that there were five cells for which the axons could not be extended owing to volume boundaries or segmentation errors, so they were replaced with the five nearest cells. On average, each of the 20 nearest neighbours had 448 synapses attached.

To quantify whether a cell preferentially targeted 5P-NP neurons, we measured the fraction of total output that targeted different predicted subclasses. Cells that output more than 30% of their synapses onto 5P-NP cells were considered to have this rare connectivity preference. A two-tailed Fisher exact test was carried out to test significance between the random cell population and the nearest neighbours.

### Predicted subclass densities

To measure the predicted cell densities per subclass across the MICrONS dataset, we divided the dataset into 50-µm^2^ bins in the *x*–*z* plane. For each bin, we calculated the number of cells in each subclass and scaled that value to the number per square millimetre to facilitate direct comparisons to reported densities in the literature.

### Dataset 2

The second dataset covers a millimetre-square cross-sectional area, and 50 µm of depth within the primary visual cortex of a P49 male mouse^18,20,51^. The largest available segmentation spans layer 2/3 of the cortex through to layer 6. After applying the nuclear detection model^18^ and filtering out all nuclear objects below 25 µm^3^ and cells that were cut off by the volume border (see the section above entitled Filtering procedure), 1,944 cells were used for the analysis. The class type of each cell was labelled manually and used as the ground truth. Owing to the thinness of the volume, much of the distal cell morphologies were cut off and thus subclass type labelling was not possible. Nuclear and somatic mesh cleaning as well as feature extraction and normalization followed the same procedures outlined above.

### Reporting summary

Further information on research design is available in the Nature Portfolio Reporting Summary linked to this article.

## Online content

Any methods, additional references, Nature Portfolio reporting summaries, source data, extended data, supplementary information, acknowledgements, peer review information; details of author contributions and competing interests; and statements of data and code availability are available at 10.1038/s41586-024-07765-7.

## Supplementary information

Reporting Summary

## Figures and Tables

**Extended Data Table 1:**
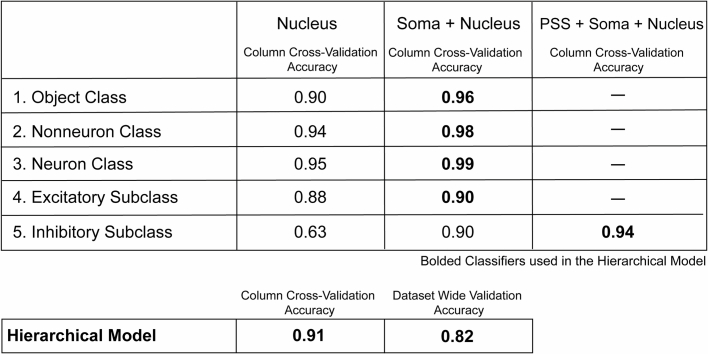
Cross validation accuracy scores for individual classifiers at each level of the hierarchical model with differing input features

Each row corresponds to the corresponding numbers in the diagram in Fig. 5a. All training examples were held consistent between features sets for appropriate model comparisons. Classifiers with the highest accuracy score at each level were included in the hierarchical model (shown in bold). The overall hierarchical model performance on the column and the dataset wide validation set (see methods) is reported at the bottom.

## Data Availability

All datasets described in the manuscript are publicly available at https://microns-explorer.org/ and https://bossdb.org/. The cell-type predictions presented in the manuscript are made available as an open-access data tool accompanying the MICrONS dataset. Users can access the most up-to-date version of these prediction via the CAVEClient; instructions can be found at https://www.microns-explorer.org/cortical-mm3. The exact predictions from this manuscript are made available in the folder https://github.com/AllenInstitute/Perisomatic_Based_CellTyping.

## Code availability

Code developed for the nucleus and soma feature extraction pipeline is available at https://github.com/AllenInstitute/Extract_Somatic_Features. Code developed for the spine and PSS extraction pipeline is available at https://github.com/AllenInstitute/featureExtractionParty/ and https://github.com/AllenInstitute/pss_extraction_pipeline. Code for the analyses presented in the manuscript is available at https://github.com/AllenInstitute/Perisomatic_Based_CellTyping.
